# Outbreaks of SARS-CoV-2 in naturally infected mink farms: Impact, transmission dynamics, genetic patterns, and environmental contamination

**DOI:** 10.1371/journal.ppat.1009883

**Published:** 2021-09-07

**Authors:** Serafeim C. Chaintoutis, Zoi Thomou, Evangelia Mouchtaropoulou, George Tsiolas, Taxiarchis Chassalevris, Ioanna Stylianaki, Maria Lagou, Sofia Michailidou, Evangelia Moutou, Jacobus Johannes Hendrik Koenen, Jacoba Wilhelmina Dijkshoorn, Dimitrios Paraskevis, Theofilos Poutahidis, Victoria I. Siarkou, Vana Sypsa, Anagnostis Argiriou, Paschalis Fortomaris, Chrysostomos I. Dovas

**Affiliations:** 1 Diagnostic Laboratory, School of Veterinary Medicine, Faculty of Health Sciences, Aristotle University of Thessaloniki, Thessaloniki, Greece; 2 Pecon Hellas PC, Dispilio, Kastoria, Greece; 3 Institute of Applied Biosciences, Centre of Research and Technology Hellas, Thermi, Greece; 4 Laboratory of Pathology, School of Veterinary Medicine, Faculty of Health Sciences, Aristotle University of Thessaloniki, Thessaloniki, Greece; 5 Pecon BV, Gemert, The Netherlands; 6 Department of Hygiene, Epidemiology and Medical Statistics, Medical School, National and Kapodistrian University of Athens, Athens, Greece; 7 Laboratory of Microbiology and Infectious Diseases, School of Veterinary Medicine, Faculty of Health Sciences, Aristotle University of Thessaloniki, Thessaloniki, Greece; 8 Department of Food Science and Nutrition, University of the Aegean, Myrina, Greece; 9 Laboratory of Animal Husbandry, School of Veterinary Medicine, Faculty of Health Sciences, Aristotle University of Thessaloniki, Thessaloniki, Greece; University of Michigan, UNITED STATES

## Abstract

SARS-CoV-2 infection outbreaks in minks have serious implications associated with animal health and welfare, and public health. In two naturally infected mink farms (A and B) located in Greece, we investigated the outbreaks and assessed parameters associated with virus transmission, immunity, pathology, and environmental contamination. Symptoms ranged from anorexia and mild depression to respiratory signs of varying intensity. Although the farms were at different breeding stages, mortality was similarly high (8.4% and 10.0%). The viral strains belonged to lineages B.1.1.218 and B.1.1.305, possessing the mink-specific S-Y453F substitution. Lung histopathology identified necrosis of smooth muscle and connective tissue elements of vascular walls, and vasculitis as the main early key events of the acute SARS-CoV-2-induced broncho-interstitial pneumonia. Molecular investigation in two dead minks indicated a consistently higher (0.3–1.3 log_10_ RNA copies/g) viral load in organs of the male mink compared to the female. In farm A, the infected farmers were responsible for the significant initial infection of 229 out of 1,000 handled minks, suggesting a very efficient human-to-mink transmission. Subsequent infections across the sheds wherein animals were being housed occurred due to airborne transmission. Based on a R_0_ of 2.90 and a growth rate equal to 0.293, the generation time was estimated to be 3.6 days, indicative of the massive SARS-CoV-2 dispersal among minks. After the end of the outbreaks, a similar percentage of animals were immune in the two farms (93.0% and 93.3%), preventing further virus transmission whereas, viral RNA was detected in samples collected from shed surfaces and air. Consequently, strict biosecurity is imperative during the occurrence of clinical signs. Environmental viral load monitoring, in conjunction with NGS should be adopted in mink farm surveillance. The minimum proportion of minks that need to be immunized to avoid outbreaks in farms was calculated at 65.5%, which is important for future vaccination campaigns.

## Author summary

SARS-CoV-2 infection outbreaks in minks were first reported on April 2020. As this condition is important for both minks and humans, we investigated natural infection outbreaks in 2 mink farms at different breeding stages. We observed a very efficient human-to-mink transmission in one of the farms, in which farmers infected a high number of minks due to blood samplings. In both farms, a wide range of symptoms was observed, followed by high mortality spanning a period of 22–23 days, indicative of massive spread among animals and between sheds due to airborne transmission. The immunity conferred after the end of the outbreak was equally very high in both farms (~93%) and epidemiological data suggest that the minimum proportion of immune minks for outbreak halting is 65.5%. The characterized SARS-CoV-2 strains possessed the mink-specific S-Y453F amino-acid substitution. Histopathological findings were suggestive of extensive lung vessel damage before the establishment of classical interstitial pneumonia lesions. We also detected SARS-CoV-2 RNA in dust and air samples after the resolution of the clinical signs. We believe that awareness should be raised for all people involved in mink handling during outbreaks, to minimize the possibilities of direct or airborne transmission through bio-aerosols or infected dust.

## Introduction

Severe acute respiratory syndrome coronavirus 2 (SARS-CoV-2) is a new member of the *Betacoronavirus* genus (*Coronaviridae* family), responsible for the coronavirus disease 2019 (COVID-19), a human disease which most likely has emerged as a spill-over from wild animals and became a pandemic through widespread human-to-human transmission [1,2]. Host tropism of the virus is determined by the binding of the viral S (spike) protein on the ACE2 (angiotensin-converting enzyme 2) receptor on target cells [3]. Besides humans, the viral receptor-binding domain recognizes ACE2 from various animal species, including species belonging in the *Mustelidae* family [4]. The susceptibility of some animal species, such as cats, to SARS-CoV-2 infection has led to natural animal infections resulting from contact between infected humans and susceptible animals [5–8].

European (*Mustela vison*) and American (*Neovison vison*) minks are members of the weasel family (*Mustelidae*). Their susceptibility, as well as their capability to transmit SARS-CoV-2 has been confirmed via experimental infection studies in ferrets [9,10] and subsequently, through outbreaks in American minks. Natural SARS-CoV-2 infection in farmed American minks was first reported on April 2020, in the Netherlands [11,12]. Minks from two separate farms displayed mild to severe respiratory and gastrointestinal clinical signs and increased mortality. Thenceforth, the number of SARS-CoV-2 infection cases recorded in minks farmed in several EU countries was high. By the end of January 2021, infections due to SARS-CoV-2 have been recorded in 400 farms located in 8 EU countries, i.e. Denmark (290 farms), the Netherlands (69 farms), Greece (21 farms), Sweden (13 farms), Spain (3 farms), Lithuania (2 farms) and France and Italy (1 farm each) [13]. This led to massive culling of affected animals and prohibition of fur farming in affected, as well as in several non-affected countries [13]. Other measures imposed included zoning, movement restrictions and strict biosecurity practices. Besides Europe, infected minks were also reported from the USA [14]. As workers of the most affected mink farms had experienced COVID-19-related symptoms prior to the outbreaks in minks, it was assumed that they provided the sources for virus transmission in the mink farms. However, recent epidemiological analyses indicate instances of multiple SARS-CoV-2 mink-to-human transmission events in mink farms in the Netherlands and in Denmark, verifying the bidirectional nature of SARS-CoV-2 transmission [11]. Most importantly, in several mink-derived SARS-CoV-2 strains, specific amino-acid substitutions were identified in the viral S protein. These substitutions are located in areas considered crucial for ACE2 receptor binding, and could also affect neutralizing antibody responses in humans [15], thus leading to reduced protection conferred by vaccine-derived or natural infection immunity from non-mutant SARS-CoV-2 strains.

Despite the previous findings from experimental infection studies in *Mustelidae* species and the extensive infections caused by SARS-CoV-2 in minks, the currently available data on SARS-CoV-2 transmissibility and the course of natural infection in farmed minks still remain scarce, as a consequence of the massive mink culling and the mink farming bans imposed [13]. On the other hand, and according to the World Organization for Animal Health (OIE), a “One Health” perspective must be implemented in order to develop epidemiological surveillance and establish control mechanisms to limit zoonotic disease transmission [16]. The global dispersal of SARS-CoV-2, and the high susceptibility of minks to the virus raises concerns that this animal species may become reservoirs of SARS-CoV-2. Additionally, their treatment on fur farms has been a focus of animal rights and animal welfare.

Taking into consideration that data regarding within-mink farm SARS-CoV-2 circulation are essential to implement optimal management practices, a field study was performed on two naturally infected mink farms located in Greece. Our aim was to investigate, first, the clinical, pathological, and epidemiological features, along with parameters associated with mink-to-mink virus transmission and herd immunity, and second, to assess the potential sources of human exposure, on occasions on which humans are required to handle minks during the entire breeding process.

## Results

### Background of the studied farms

**Fig 1:**
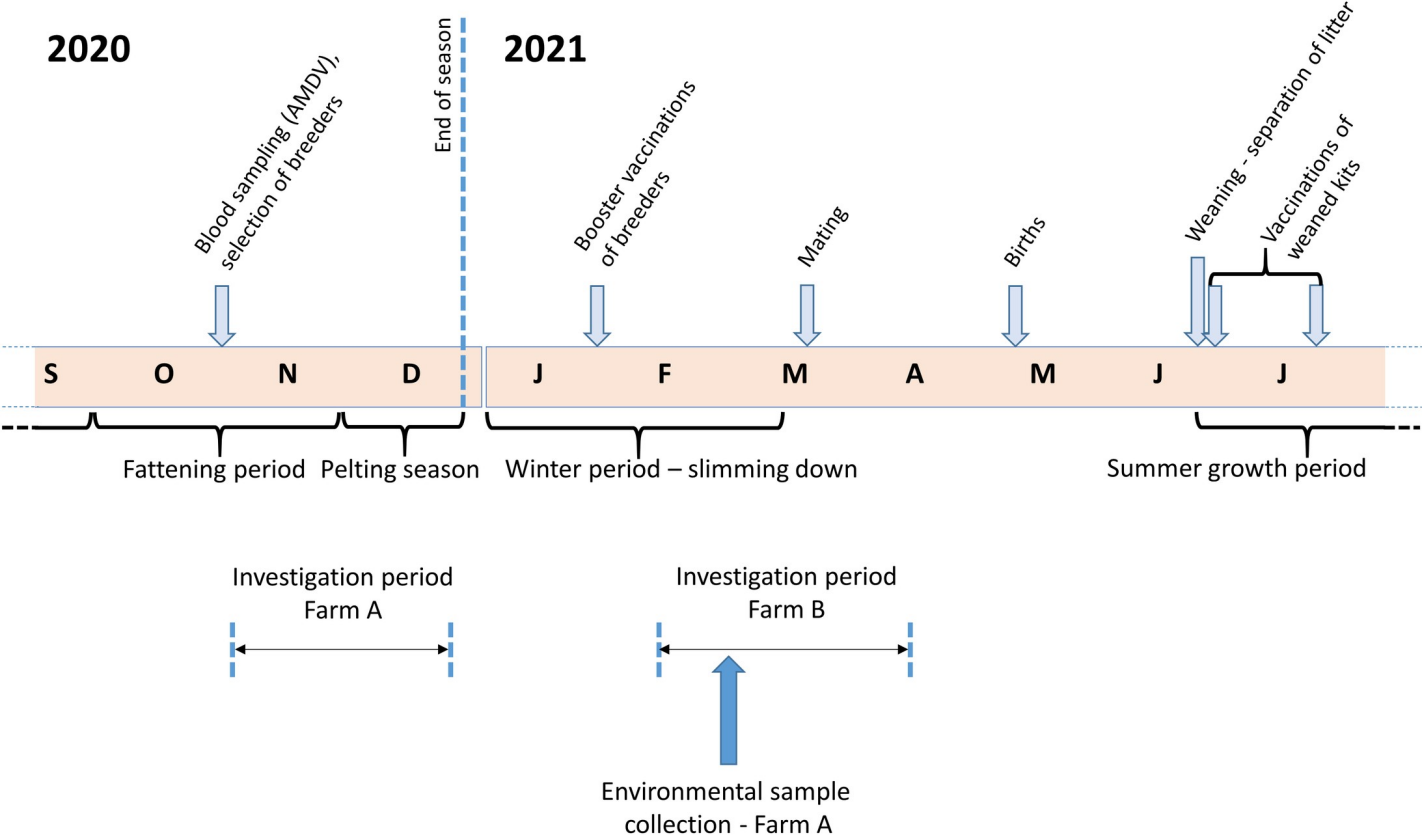
Annual cycle of mink farming and associated procedures of the breeding process. The periods wherein the farms of the present study were investigated are indicated.

The study was focused on two commercial farms (A and B) of American minks (*Neovison vison*) bred for fur production. Both farms are in the Region of Western Macedonia, Northern Greece. Specifically, farm A is located in the Regional Unit of Kozani and was studied during late 2020, whereas farm B is located in the Regional Unit of Kastoria and was studied in the first months of 2021 (Fig 1). Animals of both studied farms are *Aleutian mink disease virus* (AMDV) carriers.

**Fig 2:**
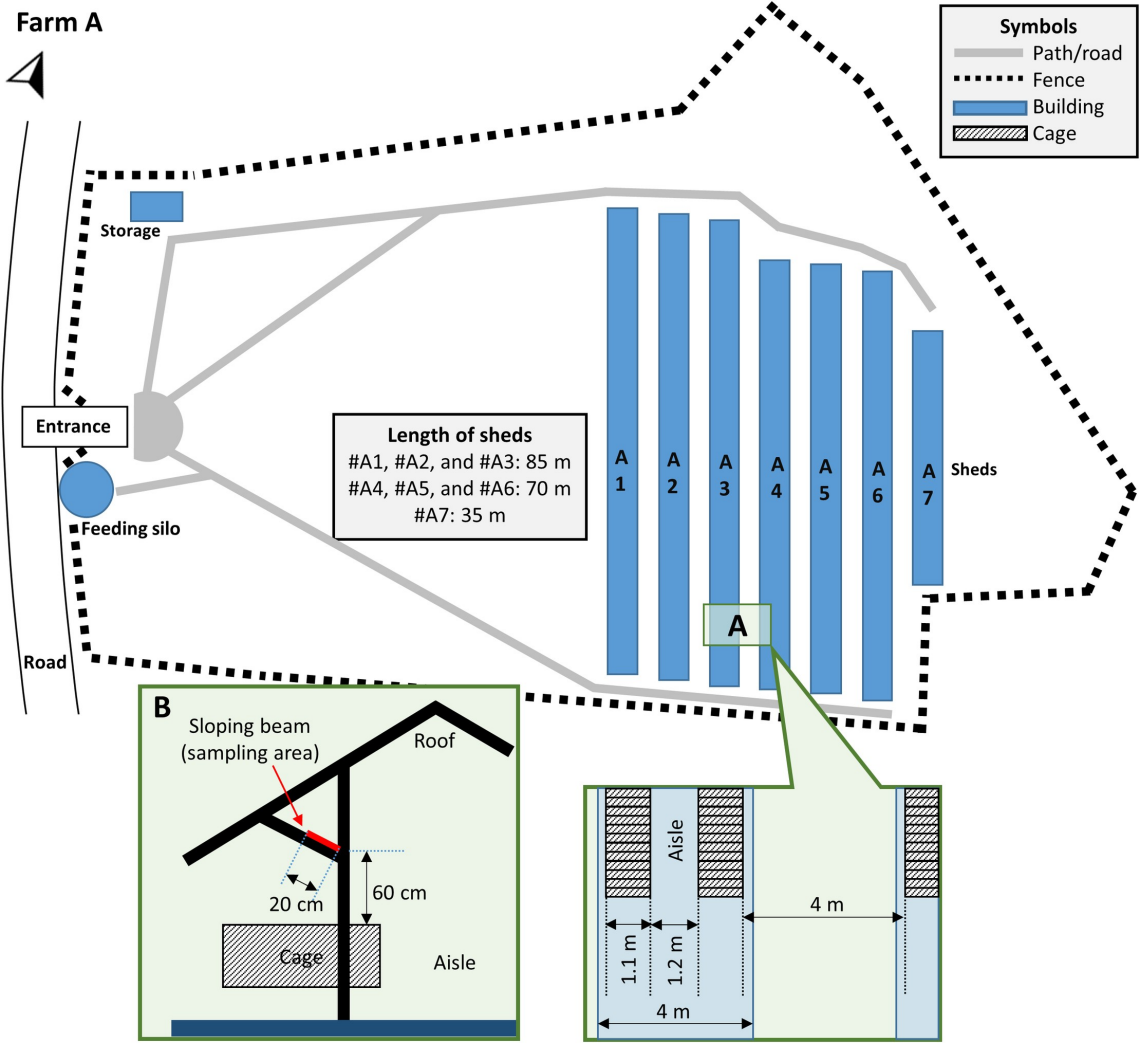
Floor plan of farm A. Inset A: floor plan detail, indicating the width of sheds, wire net cages, as well as the distances between them. Inset B: cross section of the sheds indicating the dust sampling area from the sloping beams (red marking).

**Fig 3:**
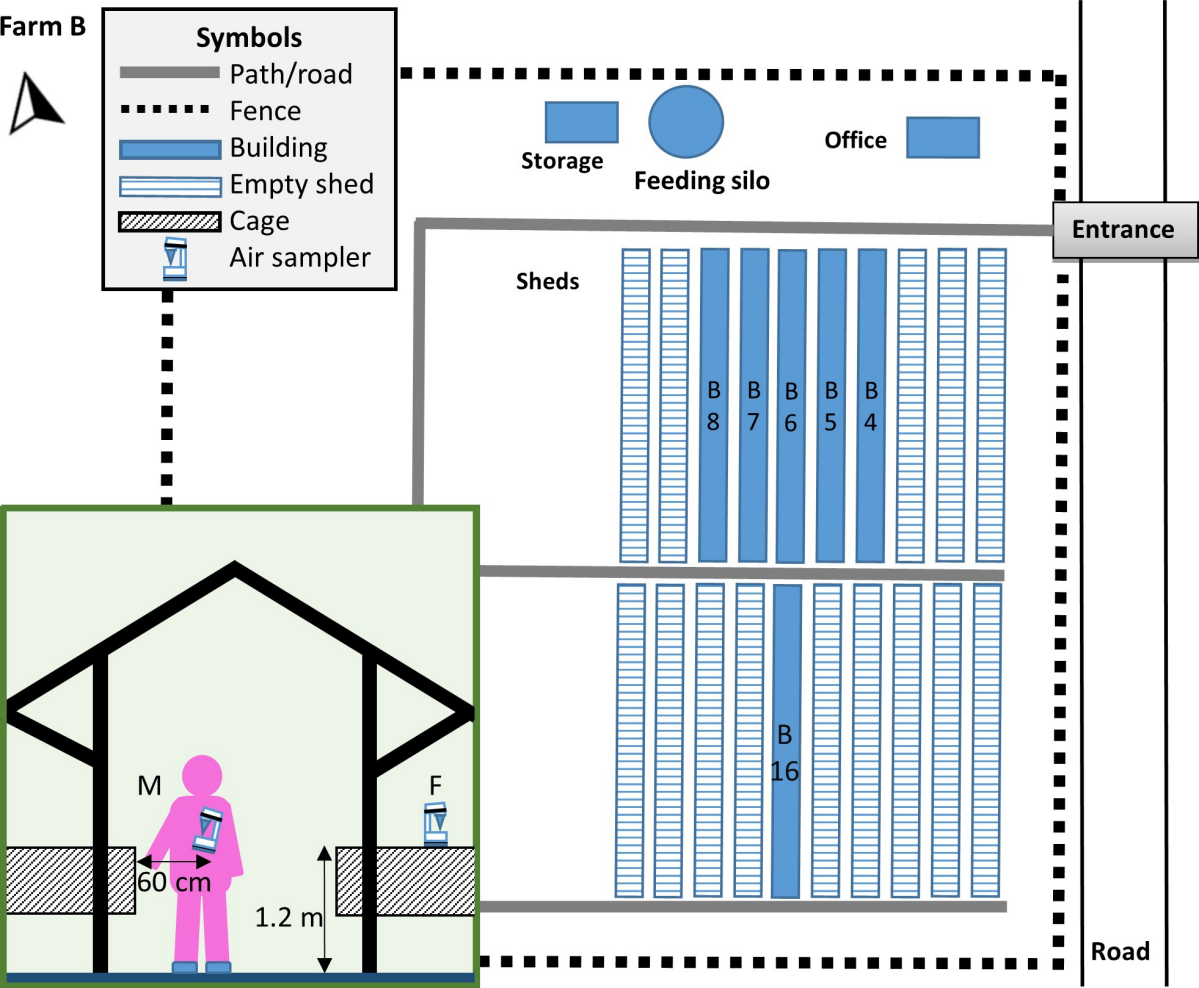
Floor plan of farm B. Animals were housed in sheds #B5-to-#B7. Sheds marked with dashed lines were empty throughout the investigation period and were not subjected to collection of environmental samples. Inset: cross section of the sheds indicating air sample collection with the microbial air sampler placed at a fixed (F) spot or being moved (M) during blood sampling.

At the time of the outbreaks, the farms were different in terms of population structure and production stage. In detail, at the beginning of its investigation, farm A had approx. 6,500 minks, and by that time, the herd had finished its growth period and was entering the pelting season (Fig 1). The minks were housed in 7 sheds adjacent to each other, in a total area of ~90 x 60 m in the farm (Fig 2). Sheds #A1-to-#A6 were full, and 20 more animals were being housed in shed #A7. Besides having a roof, the sheds are open to the wind from all sides. Each shed contains 2 rows of wire net cages, wherein animals are individually housed, and a middle aisle with a width of ~1.2 m (Fig 2). The farm is family-owned, and all procedures are being performed solely by the two farmers. Neither other people, nor vehicles are permitted to enter the farm, including any feed delivery trucks. On the contrary, farm B was studied at the beginning of the next season i.e., in early 2021 (Fig 1), and had 738 animals (breeders) housed in 3 sheds (#B5, #B6 and #B7) (Fig 3) similarly structured to those of farm A. Those animals were in the slimming down (weight loss) period (Fig 1), to achieve a suitable body condition for mating during March. Thus, their body weight was considerably reduced compared to animals of farm A, which were obese and large quantities of food were being administered to them at the time of the investigation.

**Fig 4:**
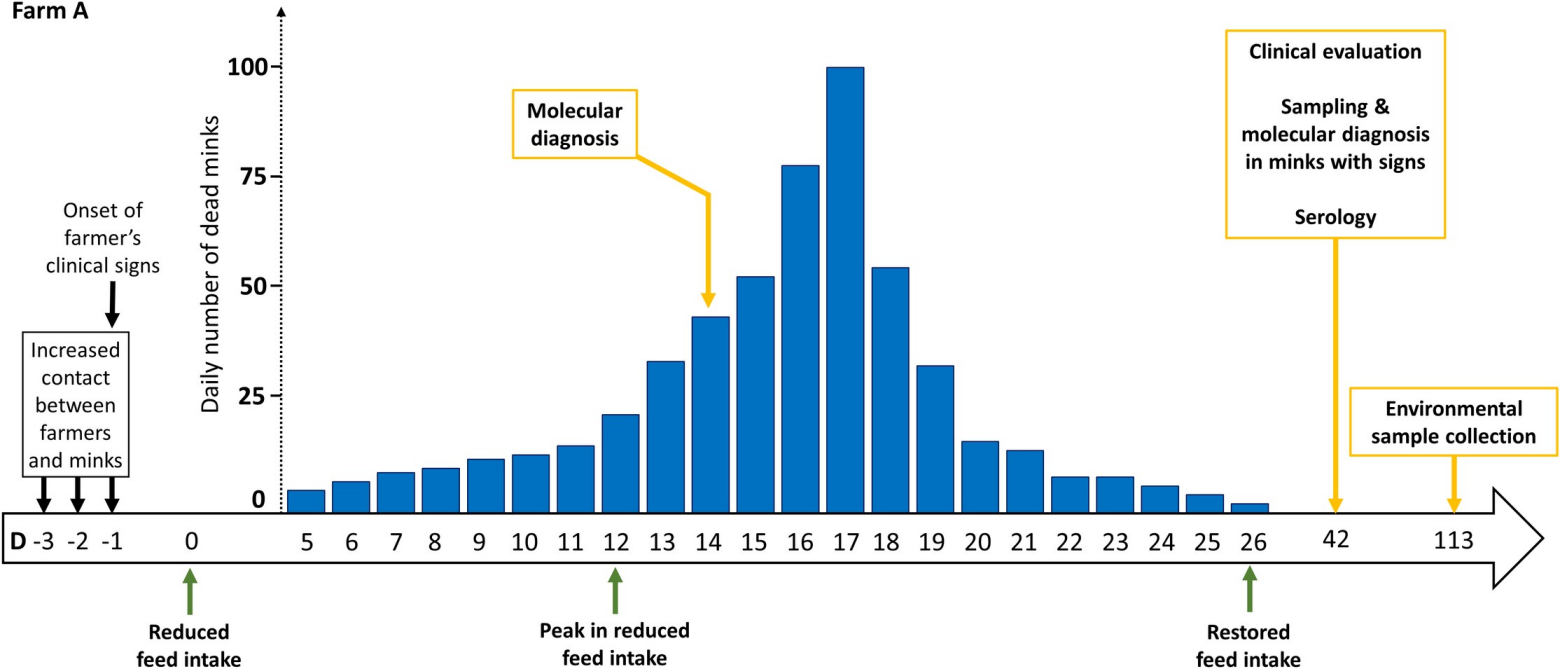
Timeline of the events observed in farm A. Yellow arrows indicate sampling procedures; green arrows refer to the clinical observations (feed intake), with the peak being associated with the highest approx. percentage of animals exhibiting anorexia at the specific time-point. The bar chart depicts the number of dead minks recorded at each time-point (day; D). Black arrows indicate blood sampling that resulted to the infection of the minks.

The outbreak in farm A started a few days after an intervention involving prolonged contact between both farmers and a high number of animals, i.e., blood sampling in the framework of AMDV serological testing. This procedure takes place before the pelting season for breeder selection (Figs 1 and 4). Samplings were initiated on day -3 (D-3) and during that day, ~500 animals of sheds #A1, #A2, and #A3 were handled, within ~5–6 hours. A similar number of animals of sheds #A4 and #A5 were sampled on D-2. The procedures were continued in shed #A5 and were expanded in shed #A6 on the following day (D-1). The farmers were not wearing masks during blood sampling. On the same (D-1) evening, after the end of blood samplings, signs of worsening fatigue, mild upper respiratory tract signs and anosmia appeared to one of the farmers. After the farmer’s symptom onset, the only farm procedures performed were feed delivery and removal of dead animals, by the second farmer who was asymptomatic. Both procedures were being performed once every day via a tractor, thus did not involve animal contact and were considerably less time-consuming (~10 min/shed). On D11, both farmers tested SARS-CoV-2-positive by a rapid antigen test, followed by real-time RT-PCR.

The outbreak in farm B could not be linked to any procedure involving prolonged contact between the farmers and the minks, as extensive animal manipulations are not required at the slimming down period. SARS-CoV-2 was not detected in the farmers and farm workers in repetitive molecular tests which were performed throughout the course of the mink outbreak (D*1, D*3, D*10, D*22), as well as in serology against SARS-CoV-2. An additional animal management practice was adopted in farm B, as animals with clinical signs were being moved to a separate, empty shed of the farm.

### Clinical observations

The clinical manifestations in minks of farm A were initiated a few days after blood samplings in sheds #A4 and #A5. As the farm was being systematically monitored by the farmers and a veterinarian, no clinical manifestations and deaths had been observed before. Signs included reduced feed intake (clinical score 1), first noticed in sheds #Α4 and #Α5 on D0 (Fig 4). During the following days, higher numbers of animals with reduced appetite were being observed, involving also shed #A6, and subsequently other sheds. Respiratory disease signs of varying intensity were also noticed. In most of the cases, these signs included nasal discharge, sneezing and coughing (clinical scores 2–3). Respiratory distress was also evident in some of the animals, with signs of abdominal breathing, noisy breathing, disorientation, and unresponsiveness to stimuli being the most severe. Animals in that phase (clinical score 4) tended to remain motionless unless it was necessary to move. The reduction in feed intake peaked on D12. Subsequently, a remission of clinical signs was evident, especially in sheds #A4, #A5 and #A6, wherein restored appetite was evident mostly. On D26, feed intake was restored in farm A. Signs of diarrhea were not observed throughout the course of the outbreak in any of the animals.

Deaths were observed in farm A, in parallel to the severe signs (Fig 4). The first 5 dead minks were recorded in sheds #A4 and #A5 (Fig 2) on D5, i.e., 5 days after the first observation of clinical signs. Deaths were also observed in shed #A6 during the next day (D6). A gradual increase was observed in deaths, which peaked on D17 with 100 dead animals. On D13, an expansion of deaths in shed #A3 was observed, and on D15 also in shed #A7. No more deaths were observed from sheds #A5 and #A6 from D18. From D20 onwards, deaths were involving only sheds #A1-to-#A3. The last 2 deaths were recorded on D26. The fraction of farm A mink population that died from the disease was 8.4%, as 548 total deaths were recorded out of the 6,500 farmed minks between D5 and D26 (i.e., 22 days).

**Fig 5:**
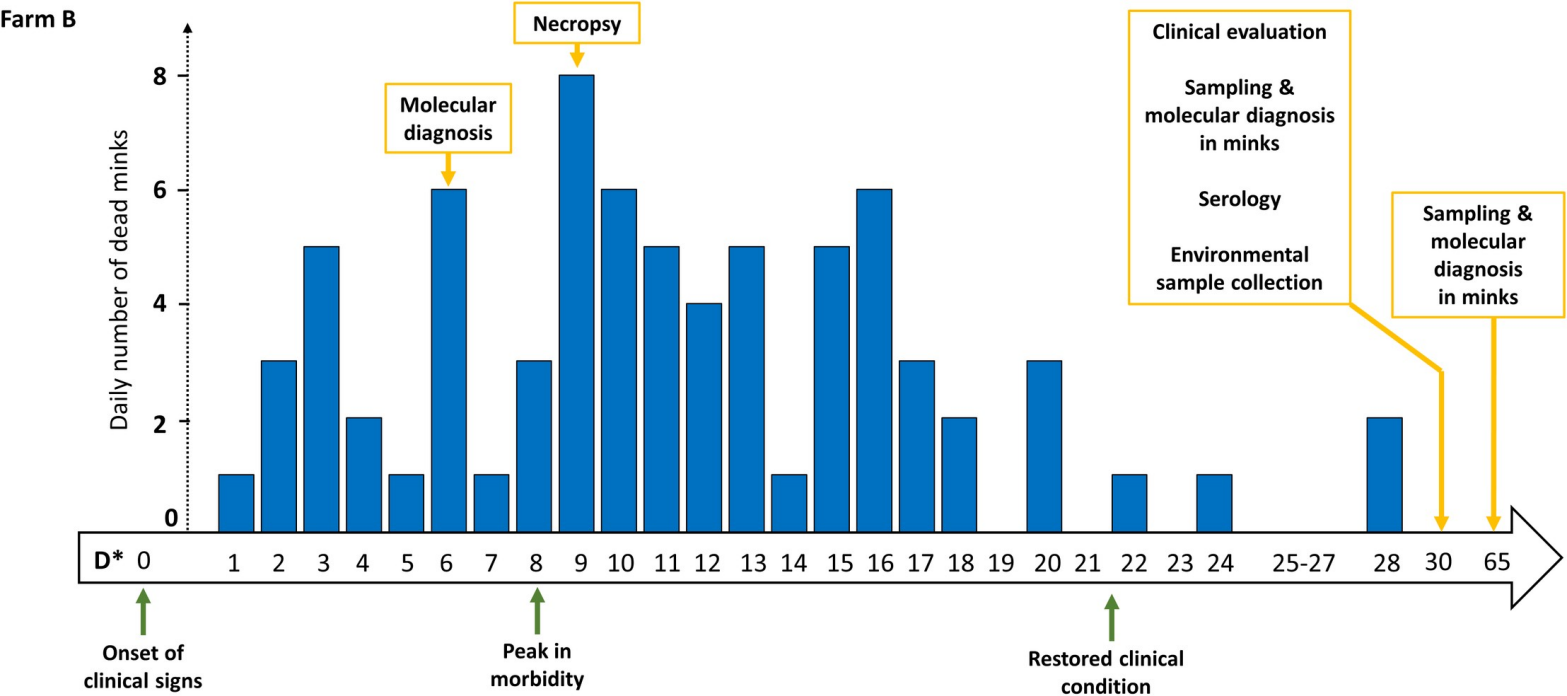
Timeline of the events observed in farm B. Yellow arrows indicate sampling procedures; green arrows refer to the clinical observations, with the peak being associated with the highest approx. percentage of animals exhibiting signs at the specific time-point. The bar chart depicts the number of dead minks recorded at each time-point (day; D*).

As animals of farm B were being administered small portions of feed (slimming down period), it was not feasible to easily recognize reduced feed intake as the first seen symptom of infected animals. For this reason, only signs corresponding to clinical scores 2-to-4 were noticed one day before the onset of deaths (i.e., D*0, Fig 5). Signs and deaths were first observed in shed #B6 and were subsequently expanded bidirectionally, to the neighboring sheds #B5 and #B7 (Fig 3). There were also days, towards the end of the outbreak, where no deaths were being recorded (Fig 5). The peak in morbidity was observed on D*8, and the number of deaths peaked on the following day (8 animals). By comparing curves of both farms (Figs 4 and 5) it is evident that the pattern of deaths in farm B was not similar to that of farm A, as the number of dead minks was fluctuating (1-to-8 per day). The fraction of farm B mink population that died from the disease was slightly higher than that of farm A (10.0%), as 74 deaths were recorded out of 738 farmed minks between D*1 and D*24.

Most of the animals that exhibited mild signs (reduced feed intake and mild respiratory signs, i.e., clinical score 1 or 2) recovered after 3–5 days. In both farms, the time from symptom onset until death was estimated for animals presenting each category of signs. Regarding animals that died, the approximate time of death was 4 days after the onset of clinical signs corresponding only to clinical scores 1 and 2. A higher percentage of animals with severe respiratory distress (clinical score 3) died, within 1-to-2 days after the onset of signs. Lastly, almost all minks with severe signs (clinical score 4) died within 1 day after their onset.

### SARS-CoV-2 detection and molecular characterization

In 10 animals from each farm presenting clinical symptoms oropharyngeal swabs were obtained (D14 for farm A and D*6 for farm B) and subjected to real-time RT-PCR-based testing for SARS-CoV-2 detection. All oropharyngeal swabs tested positive. Feces were also obtained from 6 minks (3 males and 3 females) of farm B. Virological testing revealed negative results in all 3 samples originating from females. On the contrary, all 3 samples which were obtained from males were positive, with the respective viral loads being 3.6, 5.4 and 6.2 log_10_ RNA copies/swab.

Four randomly selected real-time RT-PCR-positive oropharyngeal samples from each farm were subjected to whole genome sequencing of the SARS-CoV-2 genome to detect variants and assign possible lineages. The mink specific S protein amino-acid substitution (Y453F) was present in all the sequenced genomes (S1A Fig), as well as the ubiquitous European S-D614G substitution (Table 1). One more amino-acid substitution, the S-A879S was present only in the strains of Farm A, whereas S-V227L and S-P812L, were present in farm B (Table 1). Amino-acid substitutions were also found across other viral proteins (S1 Table). Phylogenetic analysis grouped the genomes in two distinct clades, each one corresponding to a different farm (S1B Fig). Lineages were assigned by PANGOLIN as B.1.1.218 and B.1.1.305 for farm A and B, respectively (Table 1).

**Table 1:** List of SARS-CoV-2 S protein amino-acid substitutions detected in sequenced samples from farm A and farm B. The mink-specific substitution (Y453F) is shown in bold. Lineages have been assigned with the PANGOLIN interface.

Farm	Lineage	Sample ID	Ref. position according to acc. NC_045512	Amino-acid substitution
**A**	B.1.1.218	A1-to-A4	**22920**	**Y453F**
			23403	D614G
			24197	A879S
		A2	25314	G1254V
		A2	25317	S1252C
**B**	B.1.1.305	B1-to-B4	22241	V227L
			**22920**	**Y453F**
			23403	D614G
			23997	P812L

### Pathological findings and SARS-CoV-2 viral loads in organ specimens

**Fig 6:**
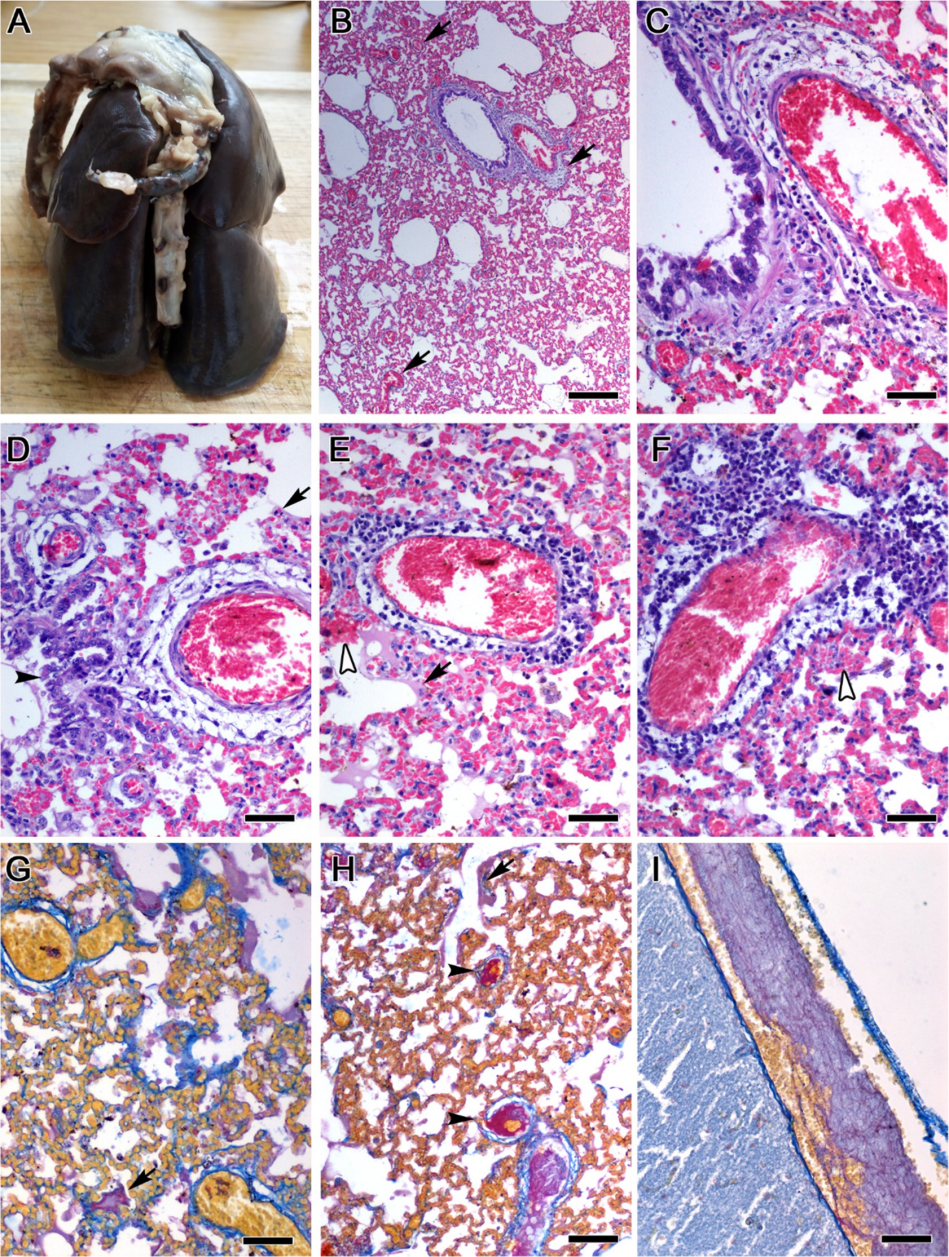
Characteristic pathological findings of SARS-CoV-2 infection in minks. (A) Formalin-fixed lung. Evidence of bilateral acute pneumonia. All lung lobes are dark brown-red in color and mildly enlarged. (B) Low-power magnification view of representative lung tissue histopathology uniformly observed. Diffuse hyperemia with vascular wall loss (arrows) and bronchus epithelium damage. (C) Part of the previous image is shown in high-power magnification to highlight vascular endothelial lining and wall destruction and edema. There is sub-epithelial edema in bronchial epithelium and degeneration and necrosis of epithelial cells. (D-F) Spectrum of inflammatory cell cuffing in damaged lung parenchymal vessels. Increasing severity of inflammatory cell infiltrate within and around the vessels from left to the right panel. Lymphocytes, plasma cells and macrophages infiltrate primarily the area of medial and external tunics that are edematous and contain necrotic cells and tissue debris. Note bronchiolar epithelial cell degeneration and necrosis (black arrow-head in D), hyaline membranes (arrows in E and F) and micro-hemorrhage from alveolar wall capillaries (white arrow-heads in E and F) and alveolar spaces with edematous fluid and alveolar macrophages. (G-I) Histochemistry highlights hyaline membranes with red (mature fibrin) color (arrows in G and H) and organized intravascular fibrin consistent with thrombus formation (black arrow-heads) in the lung (H) or the meninges of the brain (I). Hematoxylin and Eosin (B-F). Martius-Scarlet-Blue (G-I). Scale bars: 250 μm (B); 100 μm (H); 50 μm (C-G and I).

Two 10-month-old minks (one male and one female, both of farm B) that succumbed from SARS-CoV-2 infection, were necropsied at the farm and tissues were collected and histopathologically analyzed. Pathological findings involved primarily inflammation of the upper respiratory system and the lungs and fibrin thrombi formation in the lung and other tissues (Figs 6 and S2). A detailed description of all pathological findings is provided as supplementary material (S1 Appendix). It is of note that the lungs had a diffuse, acute broncho-interstitial pneumonia with prominent hyaline membrane formation and focal micro-hemorrhages in the alveolar septa but without consolidation or organizing alveolar wall damage. Severe congestion of the alveolar septa was also observed, but there was no inflammatory cell infiltration or thickening. Interestingly, vasculitis was the most prominent lesion, with endothelial cell lining and vascular wall loss and edema uniformly affecting vessels regardless of size. The spectrum of vasculitis lesions included a progressively increased in density mononuclear cell and macrophage cuffing (Fig 6).

Detection of SARS-CoV-2 RNA in the organ samples of a second pair of minks (male and female) from farm B indicated that several organs were positive (Table 2). Specifically, trachea, lung, spleen, ovary/testis and brain, along with the respective oropharyngeal swabs were SARS-CoV-2 positive for both animals, with the viral load in the male mink being consistently higher than the respective values for the female mink (difference: 0.3–1.3 log_10_ RNA copies/g tissue). Some of the organs were SARS-CoV-2 positive only in the male animal (heart, liver, pancreas and kidney; Table 2). Lastly, all gastrointestinal tract organs (stomach, duodenum, ileum and colon) from both animals were real-time RT-PCR-negative.

**Table 2:** Virological testing results of the organ samples of the two necropsied minks from farm B.

Sample	Male	Female
	Viral load (Log_10_ RNA copies/g)	
Gastrointestinal tract	Neg.	Neg.
Heart	3.7	Neg.
Liver	4.8	Neg.
Pancreas	3.7	Neg.
Testis	5.5	N/A
Ovary	N/A	4.7
Spleen	6.4	6.0
Brain	5.5	4.2
Lung	4.7	4.4
Trachea	6.0	5.0

N/A: not applicable

### AMDV serology

In both farms, > 93% of the blood samples tested for AMDV antibodies had OD values < 0.825, indicative of the absence of progressive Aleutian disease in the farms.

### Immunity, final epidemic size and infection fatality ratio (IFR) inferred from seroprevalence data

Both farms were revisited after the end of the outbreak to obtain additional samples for virus detection and serology. On the re-visit of farm A on D42 (over 2 weeks after the last death), only 7 animals out of the 5,952 minks that survived were exhibiting mild signs which raised suspicions for SARS-CoV-2 infection. Real-time RT-PCR testing of obtained oropharyngeal swabs revealed the absence of SARS-CoV-2 active infections. Serological testing of the 172 randomly selected animals sampled from farm A on D42 revealed 160 seropositive minks, with the respective seroprevalence rate being 93.0% (95% CI: 89.0%-96.5%). In three additional minks the SARS-CoV-2 serological testing result was inconclusive. Taking into consideration the 548 minks that were infected and did not survive, the final epidemic size (*z*) for farm A was estimated at 93.6% (95% CI: 89.9%-96.8%) and the IFR is estimated at 9.01% (95% CI: 8.7%-9.4%).

Farm B was revisited on D*30 and D*65. As all minks at both time-points were clinically healthy, 62 and 40 minks were randomly swab-sampled and tested, respectively. Real-time RT-PCR testing showed that no virus was detected in the samples. Similarly to farm A, serological testing against SARS-CoV-2 in farm B revealed 84 seropositive minks out of the 90 sampled i.e. a seroprevalence rate of 93.3% (95% CI: 87.8%-97.8%), which is almost identical to the respective seroprevalence rate for farm A. The result was inconclusive in two additional minks. Taking into account the number of deaths, the final epidemic size for farm B was estimated at 94.0% (95% CI: 89.0%-98.0%). The IFR is estimated at 10.7% (95% CI: 10.2%-11.3%).

### Determination of the number of minks infected by SARS-CoV-2 from humans

**Fig 7:**
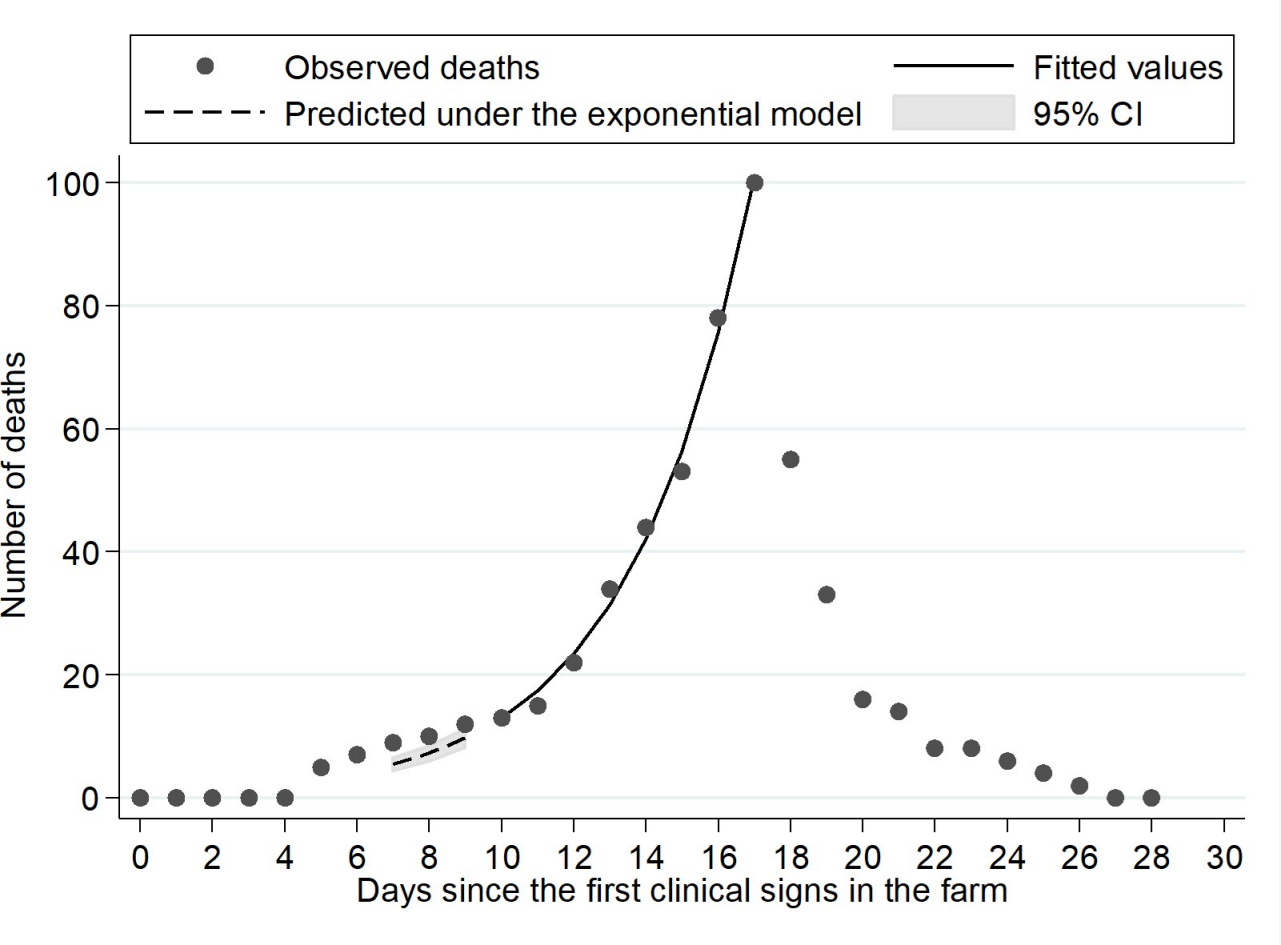
Observed number of deaths in farm A and fit of an exponential model for the days (D) 10–17. Τhe deaths observed in D5 and D6 are all attributed to initial infections from humans to minks. The difference between the observed number of deaths in D7-to-D9 and that estimated under the exponential model was used to estimate the number of deaths attributed to the initial infections from human-to-mink.

Twelve deaths were observed in farm A during D5-D6, all attributed to the initial infections from humans to minks. In addition, 31 deaths were recorded during D7-D9, a portion of which is attributed to these initial infections. An exponential model was applied to the daily number of deaths between D10 and D17 resulting from mink-to-mink transmission (Fig 7). The excess number of deaths observed during D7-D9, as compared to the expected under the exponential model, suggests that the exceeding number of deaths was due to events attributed to virus transmission from humans. The number of deaths attributed to human-to-mink transmission between D7-D9 was estimated at 8.6 (range corresponding to the 95% CI of the fitted exponential: 4.9–12.4). Considering the 12 deaths observed in D5-D6, plus the 8.6 events during D7-D9, the total number of deaths due to human-to-animal transmission was estimated to 20.6 (16.9–24.4). Based on the IFR of 9.0%, the corresponding number of animals infected by the farmers was 229 (188–271). A similar determination for farm B was not feasible, as the source of infection could not be identified.

### Epidemic growth rate and doubling time, basic reproduction number and generation time for mink-to-mink SARS-CoV-2 transmission

For farm A, the epidemic growth rate r was estimated at 0.293 per day and the doubling time was 2.4 days. Assuming that the initial fraction of infectives *ε* was 3.5% (229 out of 6,500), the estimated R_0_ was 2.90 (95% CrI: 2.51–3.52). When the lower and upper limits for *ε* was used (188 or 271 out of 6,500), the resulting estimates for R_0_ were similar (2.91 and 2.89, respectively). Based on a R_0_ equal to 2.90 and a growth rate r equal to 0.293, the generation time was estimated to be 3.6 days.

### Air and environmental contamination by SARS-CoV-2

Environmental samples (air and dust) were collected from both farms in February 2021. Air and dust testing results were presented in detail in Table 3. In the majority of the samples with a positive result, the corresponding viral load was low. The sheds with the highest mean viral RNA load in each farm were shed #A5 and #B6. These were the sheds wherein clinical signs and deaths first occurred in the respective farms.

**Table 3:** Virological testing results of the environmental samples (dust, air) obtained from the studied farms.

Farm (sampling time-point)	Type of specimen	Shed Nr. (positive/tested)	Mean [range] Log_10_ RNA copies per cm^2^ (dust) or per lit (air)	Comments on sheds and air samplings
**A** (D113)	Dust	3 (4/4)	2.22 [1.60–2.73]	H
		5 (4/4)	2.66 [2.52–2.69]	H, O
	Air	3, 5	Neg.	F
**B** (D*30)	Dust	4 (2/4)	1.47 [1.46–1.48]	S
		5 (4/4)	3.37 [2.58–3.90]	H
		6 (4/4)	3.69 [2.50–3.74]	H, O
		7 (4/4)	2.39 [1.34–2.68]	H
		8 (2/4)	1.46 [1.39–1.53]	E
		16 (0/4)	Neg.	E
	Air	5	0.33	M
		6	0.09	
		7	0.54	
		4, 8, 16	Neg.	F

F (fixed-spot): the microbial air sampler was placed on a fixed spot for sampling; M (moved): the air sampling was being performed in parallel to blood sampling and the microbial air sampler was being moved in the respective sheds; E (empty): sheds that were empty during the whole investigation period; H (housed): sheds wherein animals were being housed during the whole investigation period; O (onset): sheds wherein the onset of cases was recorded in each farm; S: empty (at the time of dust and air sampling) shed, wherein minks with signs were being isolated during the course of the outbreak.

Specifically, in farm A (Fig 2), in both air samples obtained on D113 from fixed-spots, SARS-CoV-2 was not detectable (Table 3). However, all dust samples which were collected from both sheds tested positive for SARS-CoV-2 RNA (range: 1.60–2.73 log_10_ RNA copies/cm^2^). In farm B, testing of the 6 air samples obtained on D*30 indicated that 3 out of them, which were obtained from sheds in which minks were housed (#B5-to-#B7) and the microbial air sampler was being moved, in parallel to blood sampling, were positive for SARS-CoV-2 RNA (Table 3). A positive result was also obtained for all 4 dust samples which were collected from each of these sheds (range: 1.34–3.90 log_10_ RNA copies/cm^2^). Testing of the neighboring sheds #B4 and #B8, wherein the air sampler was placed on a fixed spot, revealed a positive result in 2 out of the 4 dust samples collected from each of them (range: 1.39–1.53 log_10_ RNA copies/cm^2^). Finally, SARS-CoV-2 was not detected in any of the dust samples obtained from the adjacent empty shed #B16.

## Discussion

This study was carried out on two mink farms aimed to investigate SARS-CoV-2 transmission dynamics and the impact of SARS-CoV-2 infection in minks during the COVID-19 pandemic. Moreover, after the resolution of symptoms and deaths, we studied the immunity conferred in the surviving animals as well as the persistence/maintenance of the virus in farm environments and the possible zoonotic pathways.

Minks of farm A were infected by the farmers, during blood samplings that lasted for three days, the first of which (D-3) was performed in sheds #A1, #A2 and #A3. However, the symptoms onset (D0) was first observed in sheds #A4, #A5 and #A6, whereas cases and deaths in sheds #A1, #A2 and #A3 were detected at a later stage (i.e., 9 days after the first deaths in the farm). Thus, the time-points where minks were infected were precisely determined, i.e., D-2 and D-1, and in sheds #A4, #A5 and #A6. The fact that the sheds were open to the air from all sides and the 4 m-distance between adjacent sheds indicates that infections across the different sheds occurred due to airborne virus transmission. These findings are in agreement to a recent study that demonstrated the effective transmission of infectious SARS-CoV-2 via the air for over more than 1 m distance between ferrets [17]. Clinical signs were first observed in sheds #A4 and #A5 two days after infection of the minks. It is expected that in parallel with the onset of signs, infected minks were emanating high viral loads, leading to a mink-to-mink virus transmission chain. This has also been supported by experimental infection studies in ferrets (also a member of *Mustelidae* family) [10,18] and also in a recent experimental study in minks [19] indicating viral shedding 48 h after virus inoculation. As a result, not all deaths are associated with mink-to-mink transmission, as 20 deaths (3.67% of the total deaths) were due to the initial infection.

Comparison of the temporal distribution of deaths between the two studied farms indicates that a bell-shaped epidemiological curve was not observed in farm B, in contrast to farm A. Additionally, the fraction of the mink population dying from the disease in farm B was slightly higher compared to that of farm A, and towards the end of the outbreak there were days wherein no deaths were recorded. These observations can be attributed to differences between the two farms, i.e., the total number of farmed minks in each of them, factors associated with: i) the different fur production stage, ii) the significant initial infection of minks in farm A which did not occur in farm B, as well as iii) the fact that, in farm B, animals with clinical signs were being moved to a separate shed. However, the serological study which was conducted after the resolution of the outbreaks revealed similar percentages of seropositive animals. These high seroprevalence rates, along with the fact that SARS-CoV-2 circulation was halted at similar time-points, are indicative of the establishment of herd immunity after the rapid and extensive SARS-CoV-2 transmission among the farmed minks. Τhe estimated R_0_ in farm A, along with the high growth rate of the epizooty, indicate the massive rate of SARS-CoV-2 dispersal among mink. Moreover, the estimated herd immunity indicates that is the minimum proportion of minks that need to be immune for transmission to be halted was 65.5%. This is of value for future vaccination programs, to avoid SARS-CoV-2 transmission in farmed minks.

In the advent of the new vaccines and given that the target for immunization is the viral spike protein, mutations may alter its structure and thus, vaccine efficacy against those variants may be decreased. Thus, viral genome sequencing should be adopted in the framework of mink farm surveillance protocols to identify variants and also, to determine the directionality of transmission between humans and minks [11]. To detect possible emerging variants, we sequenced eight SARS-CoV-2 genomes from positive mink samples. Despite the assignment to the lineages B.1.1.218 and B.1.1.305, additional polymorphisms from those described in the PANGOLIN rules for these two lineages, were detected in the sequenced genomes from both farms and particularly in the spike protein. Most single-nucleotide polymorphisms (SNPs) found within the S gene were mostly accumulated in the spike protein RBM region [20], with the mink-specific amino-acid substitution (S-Y453F) being present in all sequenced genomes. Although more data are needed to elucidate the evolution of SARS-CoV-2 variants in minks, the independent presence of Y453F in Greek mink farms suggests its positive selection for mink-specific mutations.

To our knowledge, this is the first quantitative description of the SARS-CoV-2 circulation outcome and herd immunity in SARS-CoV-2 affected mink farms. It should be noted that both studied farms were also seropositive to AMDV, which is common for mink farms. However, progressive Aleutian disease was not observed in any of the farms studied herein, thus the implication of AMDV in the clinical occurrences observed is disputed. It would be interesting in the future to investigate the clinical and epidemiological features of SARS-CoV-2 infection in AMDV-seronegative farms. To further investigate the pathogenetic mechanisms associated with the SARS-CoV-2-related disease in minks, a small number of animals from farm B was necropsied and several organs was examined histopathologically.

Pathological findings in the upper respiratory tract and the lungs at large match recently reported findings in naturally [12,21], or experimentally infected minks [19]. They are also consistent with findings described in experimental infections of ferrets [10], golden Syrian hamsters [22,23] and primates [24,25]. One important difference, however, is that in the case of the two minks examined in the present study, there was no evidence of lung consolidation or accumulation of inflammatory cells in the alveolar septa. The most obvious explanation for this discrepancy is that the minks examined here succumbed during an early phase of the acute broncho-interstitial SARS-CoV-2-induced pneumonia, when the classical interstitial pneumonia finding of inflammatory cell infiltration of alveolar walls is not yet evident. Instead, we find that, in this early phase, inflammatory cells accumulate around damaged vessels. Thus far, vessel wall necrosis with vasculitis and perivasculitis as the one described in the present study has not been reported in naturally-infected minks [12,21]. However, in experimentally-infected minks Shuai et al. have described fibrinoid necrosis of vascular wall and severe lymphoplasmacytic perivasculitis and vasculitis [19]. Likewise, others report perivascular infiltrates of small numbers of lymphocytes in the lung vessels of experimentally-infected rhesus macaques [25] and lymphoplasmacytic perivasculitis and vasculitis in the lung vessels of experimentally-infected ferrets [10]. The lung histopathological analysis indicates that vascular wall smooth muscle and connective tissue element damage was an important early event in SARS-CoV-2-induced acute pneumonia of the two minks examined. Taken together with recent results of experimental infections of minks, primates and ferrets [10,19,25] these data suggest that the possibility of a direct, early SARS-CoV-2 cytopathologic effect in cells comprising the contractility apparatus of vessels, namely the vascular wall, warrants further investigation. This is not completely improbable, since smooth muscle cells of vascular walls express the ACE2 receptor [26]. This would also parallel other coronavirus-induced diseases of veterinary importance, such as feline infectious peritonitis in which fatal pathology initiates as pyogranulomatous vasculitis [27].

Upper respiratory tract pathology in trachea and nasal mucosa found in the present study have been described in SARS-CoV-2 infection of animals [10,21–23]. Also, the presence of intravascular fibrin or thrombi found in the lung or other organs including the brain is a common finding in both experimentally minks [19] and humans [28–31]. Several lesions found in other organs, such as thymus and ileum, however, may be incidental and cannot be directly linked with SARS-CoV-2 infection, based on the examination of just two animals. Gender-related differences in the course and outcomes of SARS-CoV-1 and SARS-CoV-2 infections have been documented in humans as well as in laboratory animals. Infection of mice with SARS-CoV-1 resulted in enhanced susceptibility, associated with elevated virus titers, enhanced vascular leakage and alveolar edema [32]. It has been also documented that men with COVID-19 are more at risk for worse outcomes and death, independent of age [33]. It has been also hypothesized that the male reproductive system could be a potential target of SARS-CoV-2 infection, and could also lead to infertility [34]. To our knowledge, such differences in viral load and infection outcome between male and female minks have not been documented so far. Virological testing of the organs obtained from the second pair of minks (male and female) indicated a consistently higher viral load in the organs of the male mink compared to those of the female mink, whereas there were also organs which were positive only in the male mink. All gastrointestinal tract organs were SARS-CoV-2-negative in both animals. However, testing of feces which were obtained from 6 animals revealed a positive result in 3 of them, which were also male. The results obtained herein are of use for future studies aiming to quantify the gender-related differences in minks as well, as possible implications of the infection in the fertility of male minks. Additionally, the fact that fecal samples tested positive is indicative of the fact that, besides infected bio-aerosols, feces from infected minks also contribute to the increase of the environmental viral load in affected farms.

SARS-CoV-2 RNA was detected in dust samples collected from both mink farms, as well as in air samples collected from farm B. Interestingly, SARS-CoV-2 was detected in dust of farm A two months after the resolution of the outbreak. Besides, the infectivity of the detected virus in the dust obtained from farm A is not expected, as the viral load was low, and the detected viral RNA had been present at the environment for over 60 days. The prolonged presence of SARS-CoV-2 RNA in dust samples is possibly indicative of the high loads being emanated from infected minks during their infection course. In addition, the fact that dust was obtained at heights >60 cm over the top of the cages further signifies the magnitude of virus transmission via infected respiratory bio-aerosols. The contamination of surfaces by SARS-CoV-2 genetic material has been previously demonstrated at various settings [35–37]. Mink-to-mink transmission in farms occurs through contaminated dust from the excretions of the infected animals, including fecal matter. It has also been suggested that SARS-CoV-2-positive inhalable dust may act as a potential source of infection for the farmworkers [12], and therefore, the staff implicated in mink farm procedures is exposed to risk of SARS-CoV-2 infection [38]. Even though the personnel of farm B tested negative in repetitive samplings for SARS-CoV-2 and specific antibodies, potential inhalable contaminated dust, whenever minks must be handled by farmworkers, may contribute to zoonotic SARS-CoV-2 transmission, highlighting the need for strengthening biosecurity measures.

It is known that, at ambient air temperatures (i.e. around 20°C), coronaviruses persist for a few days, depending on the matrix, temperature and relative humidity [39]. It has been also shown that the stability of SARS-CoV-2 on various surfaces varies, with a 3 log_10_ infectivity loss after 48 h or 72 h on stainless steel and plastic, respectively [40]. However, Riddell et al. demonstrated that infectious SARS-CoV-2 can be recovered from non-porous surfaces for at least 28 days at ambient temperature and humidity [41]. The fact that the environmental temperatures during the investigation period in farm A (November-to-February) were extremely low, may have favored the preservation of the viral nucleocapsid. Given that the serological testing conducted in farm A revealed that 7% of the animals were naïve, along with the observation that SARS-CoV-2 transmission was halted, advocate for reduced chances that the contaminated dust was infective. In our case, SARS-CoV-2 isolation from environmental samples was not performed, thus further research is required to clarify the infectivity of SARS-CoV-2 on contaminated environmental surfaces in farms. The fact that higher titers of SARS-CoV-2 RNA were observed in sheds wherein the clinical signs and deaths were initiated is of importance and should also be investigated in the future, as dust sampling using wipes comprises a convenient approach to monitor the environmental viral load and the magnitude of viral shedding in affected farms, with applicability for epidemiological investigations.

In conclusion, the findings of the present study demonstrate that SARS-CoV-2 is highly transmissible from humans to minks. The mink-to-mink spread course in farms is massive and a high fraction of the animals dying from the disease is observed, irrespective of the different breeding stages. Lung histopathology results indicate the possibility that the virus damages extensively the vessels of the lung before classical interstitial pneumonia lesions establish. This may lead to the conclusion that COVID-19 is primarily a vasculopathy, thus affecting the therapeutic strategies. During the outbreak, the high viral loads emanated from infected minks led to environmental contamination, and strict biosecurity measures are imperative. After the resolution of the outbreaks, further virus circulation is halted because of the established herd immunity, which is reached within 24–28 days. Decontamination of surfaces could also be applied at this time point, as an additional biosecurity measure, for efficient and rapid reduction of viral loads. Lastly, viral load monitoring from surfaces in conjunction with Next-generation (NGS)-based variant identification should be adopted in farm surveillance to avoid possible spillover of SARS-CoV-2 variants from animals to humans. Finally, the data related to herd immunity presented herein suggest that SARS-CoV-2 transmission halting in mink farms can be achieved by vaccinations, even in cases where very high percentages of immunity are not achieved.

## Materials & methods

### Ethics statement

The authors confirm that the ethical policies of the journal, as noted on the journal’s author guidelines page, have been adhered to. No ethical approval was required, as anonymous data were used, all animal samples were taken for diagnostic purposes in the framework of non-experimental clinical veterinary practices and no medical interventions were made on animal or human subjects.

### Sampling for virus identification and serology

After the observation of the clinical signs (D0 for farm A, D*0 for farm B) and the increasing numbers of dead minks, the national veterinary authorities were notified, and the farms were visited by veterinarians for oropharyngeal swab sampling. In each farm, ten suspected randomly selected minks were subjected to sampling for molecular diagnosis. Specifically, oropharyngeal swabs were collected using the CITOSWAB collection kit (VTMK-49-3ML), in accordance with CDC recommendations for the collection and handling of human clinical specimens.

Reduced feed intake was evident through the observation of feed leftovers at the cages. The range of symptoms and their severity, as well as the approximate percentage of animals presenting signs were estimated. It was not feasible to determine the exact numbers of animals that displayed symptoms each day. However, the daily number of dead minks were recorded. Additionally, the observed clinical signs were roughly scored on a 4-point scale (clinical scores; 1 = reduced feed intake, 2 = mild respiratory signs, 3 = severe respiratory signs, 4 = abdominal breathing or lethargy and unresponsiveness to stimuli) and the approximate time needed for death was estimated for animals presenting each scoring category.

Both farms were re-visited over 2 weeks after the resolution of symptoms and the last recorded death. At that time-points (D42 for farm A; D*30 and D*65 for farm B) thorough clinical evaluations were performed. All animals with clinical signs that could be attributed to SARS-CoV-2 infection underwent oropharyngeal swab sampling for real-time RT-PCR testing. At the same time-points, blood samples were randomly collected by toenail clipping from both farm A (n = 172 animals) and farm B (n = 90 animals) for serology, to estimate the levels of established immunity in each of the studied farms. The sampling size for blood sera was calculated through the StatCalc function of the Epi Info software (CDC, Atlanta, USA), using a 95% confidence level.

### Fecal samples

Fecal samples were collected from 6 animals from farm B (3 male and 3 female). After sample collection, fecal samples were placed in virus-inactivating guanidinium isothiocyanate-based “Lysis buffer I” [42].

### Organ samples

In farm B, four minks (2 male and 2 female) that died from typical SARS-CoV-2 infection respiratory symptoms were subjected to necropsy on site, shortly after their death (D*9). Organs from one pair of minks (male and female) i.e., lungs, trachea, heart, nasal mucosa, liver, spleen, kidneys, adrenal glands, brain, stomach, duoedenum, jejunum, ileum and colon were collected and stored in 10% buffered formalin. Formalin-fixed tissues were routinely processed, embedded in paraffin, cut at 5 μm, and stained with hematoxylin and eosin or the Martius-Scarlet-Blue (MSB) histochemical stain for fibrin. Organ samples from the second pair of minks (male and female) were obtained for SARS-CoV-2 molecular detection and were processed for virological testing with the same protocol as fecal samples.

### Environmental samples

Farm A was re-visited on D113, for collection of environmental samples, i.e., dust and air. Samples were collected from sheds #A5 and #A3 using 2 separate approaches; Dust samples were collected using electrostatic dust wipes (Swiffer; Procter and Gamble, USA), which were aseptically pre-cut in round discs with a diameter of 4.5 cm. Within each shed, four different sloping beams located >60 cm above the cages were sampled. Using sterile forceps, discs were placed on the respective sampling area with the electrostatic surfaces facing downwards and dragged onto the beam surface for 20 cm. Air samples were obtained from the sheds using the Coriolis Compact microbial air sampler (Bertin Instruments, Montigny-le-Bretonneux, France). In each shed, the instrument was placed at a height of 1.2 m and run for 25 min, which corresponds to sampling of 1,260 lt of air. This volume of air equals the total volume of air inhaled by a human staying in the shed for a period of 6 h. The dust collection discs, as well as the inner surfaces of the cyclonic collector cones were rinsed with virus-inactivating “Lysis buffer I” [42].

Dust and air samples were also collected from farm B on D*30, as described. Six sheds (#B4-to-#B8 and #B16, Fig 3) were sampled and 4 dust samples were obtained per shed. In 3 of the sheds (#B5, #B6, and #B7), air was sampled in parallel with blood sampling from the minks, with the device being placed as close as possible to the person performing the blood sampling. In this case, the air sampler was not placed at a fixed spot, but was being moved in the shed, and kept at a horizontal distance of ~60 cm from the cages. The height was the same (1.2 m) as in all other sheds of farm B and farm A, in which the air sampler was placed at a fixed spot.

### RNA extraction and SARS-CoV-2 real-time RT-PCR assays

All mink oropharyngeal swabs were subjected to RNA extraction using the MagMAX Viral/Pathogen Nucleic Acid Isolation Kit (Thermo Fisher Scientific, USA) on the automated KingFisher Flex Magnetic Particle Processor (Thermo Fisher Scientific), under the manufacturer’s instructions. Real-time RT-PCR testing was performed on a Rotor-Gene Q 5plex Platform (Qiagen), using the PrecisionPLUS OneStep RT-qPCR Master Mix (Primer Design, UK) and a combination of primers and probes targeting the RdRp gene (nCoV_IP4-14059Fw, 14146Rv and 14084Probe(+); Institut Pasteur, France) and S gene [43] (Joint Research Centre, European Commission), respectively.

Organ samples, fecal swabs and environmental samples underwent RNA extraction using a phenol-chloroform-based RNA extraction process coupled with silica column binding [42]. Real-time RT-PCR testing in those samples was performed using the N2 protocol proposed by the Centers for Disease Control and Prevention (CDC) for the diagnosis of COVID-19 in humans [44]. All oligonucleotides (primers and TaqMan probes) were synthesized by Integrated DNA Technologies (IDT, Coralville, IA, USA). The assay was performed on a CFX96 Touch Real-Time PCR Detection System (Bio-Rad Laboratories, Hercules, CA, USA). Analysis of fluorescence data was performed using CFX Maestro software (v4.1; Bio-Rad Laboratories, Hercules, CA, USA). RNA extracts with a Ct value > 40 were considered as negative. Calibration curves were also generated for virus quantification, using the synthetic single-stranded RNA standard “EURM-019” (Joint Research Centre, European Commission. Virus titers in organ samples were expressed as log_10_ SARS-CoV-2 RNA copies per gr of tissue (organs), per swab (feces), per lit. of air (microbial air sampler cones) or per cm^2^ of sampling area (dust sampling discs).

### NGS and SARS-CoV-2 variant detection

In the two under study farms real-time RT-PCR-positive mink samples (four per farm) were subjected to whole SARS-CoV-2 genome sequencing. Ten microliters of RNA extract underwent cDNA synthesis, using the SuperScript II reverse transcriptase (Thermo Fisher Scientific) and 50 ng/μl of random primers, according to the manufacturer’s guidelines. For NGS library preparation, the ARTIC v3 protocol developed by Wellcome Sanger Institute was used [45], with minor modifications. Specifically, for cDNA amplification using the ARTIC PCR primer pools (v3), 2.5 μl of the generated cDNA was used instead of 6 μl. Finally, the NEBNext adaptor (NEB #7335), used in the ligation reaction, was diluted at a final concentration of 10 μΜ. All purification steps were performed according to the ARTIC protocol using Agencourt AMPure XP beads (Beckman Coulter) and the DNA concentration measured by the Qubit 4 Fluorometer (Invitrogen) using the Qubit dsDNA BR Assay Kit (Invitrogen). NGS reactions were run on an Illumina MiSeq System (Illumina, USA), with a read length of 2 × 300 bp.

Variant discovery was performed with the GATK4 set of tools. In detail, the raw reads were filtered for quality and remaining adapter sequences with Trim Galore! v0.6.5. Filtered reads then mapped to the reference viral genome (RefSeq Assembly: GCF_009858895.2) using MiniMap2. HaplotypeCaller was used for variant calling. The filtration of the variants performed with VariantFiltration, SelectVariants, BaseRecalibrator, ApplyBSQR and BaseRecalibrator tools in the order that were mentioned. The bcftools consensus was used to extract the consensus sequence in fasta format. Lineages were assigned via the PANGOLIN SARS-CoV-2 lineage assigner interface [46] and variant annotation performed with SnpEff [47].

Multiple sequence alignment and phylogenetic analysis were performed using Mega X [48]. The evolutionary history was inferred using the Neighbor-Joining method [49] using the Mega X standard parameters. The evolutionary distances were computed using the Maximum Composite Likelihood method and are in the units of the number of base substitutions per site. All ambiguous positions were removed for each sequence pair (pairwise deletion option).

### SARS-CoV-2 serology

The commercially available ID Screen SARS-CoV-2 Double Antigen Multi-species ELISA (ID.vet, Montpellier, France) kit was used, under the manufacturer’s instructions, for the detection of antibodies directed against the N protein of SARS-CoV-2.

### Investigation of AMDV infection status

Since both studied farms were seropositive to AMDV, it was considered important to evaluate whether there was progressive Aleutian disease. The blood samples obtained from both farms were assayed by a commercially available ELISA kit (AMDV Antibody ELISA Test, Reference ADV3005, Scintilla Development Company LLC; Bath, Pennsylvania, USA) as previously described [50] according to the manufacturer’s recommendations. Samples with OD values < 0.825 [51] are indicative of absence of progressive disease.

### IFR estimation

IFRs were estimated in both farms as the proportion of deaths among all infected animals. We estimated the proportion of infected animals from testing N = 172 (farm A) and N = 90 (farm B) animals out of those still alive at the end of the outbreak (SARS-CoV-2 ELISA). Thus, the total number of infected animals was determined after drawing values for the proportion infected out of those alive at the end of the epidemic using a binomial distribution and including the total number of deaths.

### Transmission dynamics

In farm A, the basic reproduction number R_0_ for mink-to-mink transmission was estimated. We assumed that an initial number of animals were infected directly from the farmers during blood samplings and that subsequently there was no human-to-mink transmission as the procedures performed from D0 and onwards involved no animal contact and lasted for a short period of time (~10 min/shed). The following formula was employed to estimate the basic reproduction number R_0_ [52]:

$$R_{0}=\frac{\left(1-z)-log(1-\varepsilon \right)}{-z}$$

where *ε*: the initial fraction of infectives, i.e., the fraction of animals infected directly from humans and *z*: the final epidemic size.

The initial fraction of infectives *ε* was obtained by determining the number of deaths occurring in the first days that could be attributed to the animals infected by the farmers and applying the infection fatality ratio to calculate the number of these infections. The first deaths were recorded in sheds #A4 and #A5 on D5, whereas in shed #A6 on D6, approximately 7 days after the respective blood samplings were performed and significant initial infection from human-to-mink occurred. Thus, a minimum 7-day time interval from infection to death can be inferred. Assuming viral shedding in minks 48 hours after virus inoculation, the deaths that occurred on D5 and D6 can all be associated with human-to-mink transmission. As an exponential increase in the number of deaths since D10 was observed, reflecting the increase in the number of cases from mink-to-mink transmission, we assumed that a portion of deaths during D7-D9 were attributed to the initially infected minks. Thus, we fitted an exponential model to the observed number of deaths recorded from D10 to D17 and predicted the anticipated number of deaths during D7-D9. The excess deaths, i.e., the difference between the observed number of deaths and that anticipated under the exponential model (upper and lower 95% CI), was attributed to infections from humans.

The final epidemic size z is the fraction estimated using the total number of minks in farm A as the denominator and the cumulative number of infected animals by the end of the outbreak as the numerator. Thus, the final epidemic size was estimated after drawing values for the proportion infected out of those alive at the end of the epizooty using a binomial distribution and including the total number of dead animals. This was repeated 10,000 times and the median as well as the 2.5^th^ and 97.5^th^ percentiles are provided for z and the resulting R_0_. We further estimated the epidemic growth rate r from the exponential model fitted to the daily number of deaths, the doubling time *d*_*t*_ as ln(2)/r and the generation time *T*_*g*_ as ln(R_0_)/r.

## Supporting information

S1 Appendix Detailed description of all pathological findings in two necropsied minks. (DOCX)

S1 Table List of amino-acid substitutions detected in the SARS-CoV-2 strains of farm A and farm B, excluding those of the spike protein. (DOCX)

S1 Fig (A) Multiple alignment of the S protein amino-acid sequences with MUSCLE v3.8. The mink-specific Y453F amino-acid substitution is indicated. (B) Phylogenetic analysis of the sequenced samples conducted with MEGA X. The evolutionary history was inferred using the Neighbour-Joining method. The percentage of replicate trees clustered together in the bootstrap test (500 replicates) are shown next to the branches. The evolutionary distances were computed using the Maximum Composite Likelihood method and are in the units of the number of base substitutions per site. The lineage assigned by PANGOLIN is indicated with brackets.
(TIF)

S2 Fig (A) Trachea mucosa with hyperemia, lamina propria edema and respiratory epithelial changes including loss of cilia and degeneration and necrosis of epithelial cells. (B) Nasal conchae respiratory epithelium showing flattening of epithelial cells and loss of cilia, and abundant necrotic-cell rich, suppurative exudate in the cavity. (C and D) Ileum mucosa with loss of germinal center lymphocytes in the submucosal lymphoid tissue (C) and goblet cell hyperplasia (D). (E and F) Thymus medulla histiocytosis (E). There is a large number of macrophages in various stages of red blood cell phagocytosis (F). Hematoxylin and Eosin. Scale bars: 100 μm (C and H); 50 μm (D); 25 μm (A, B and F).
(TIF)
