# Supplementary material for: Outbreaks of SARS-CoV-2 in naturally infected mink farms: Impact, transmission dynamics, genetic patterns, and environmental contamination
Source: PLoS Pathog. 2021 Sep 7;17(9):e1009883. doi: 10.1371/journal.ppat.1009883 (PMC8448373; doi:10.1371/journal.ppat.1009883)
Supplement: S1 Appendix — (DOCX) [file ppat.1009883.s001.docx]

**Pathological findings**

Two 10-month-old minks (one male and one female, both of farm B) that succumbed from SARS-CoV-2 infection, were necropsied at the farm. They had similar gross pathology. The lungs were diffusely dark red in color and mildly enlarged without areas of frank consolidation. The trachea had a diffusely congested mucosa and contained small amounts of frothy material suggestive of mild-to-moderate pulmonary edema. The nasal cavity mucosa and the turbinates were congested and contained a mucopurulent exudate. The remaining organs apart from being moderately congested had no grossly visible lesions.

The histopathological examination of the formalin-fixed lungs (Fig 6A) revealed comparable histopathological findings in the tissues of both the male and the female animal. Diffuse, acute broncho-interstitial pneumonia, without consolidation or organizing alveolar wall damage was present. Severe congestion of the alveolar septa was also observed, but there was no inflammatory cell infiltration or thickening (Fig 6B). Vasculitis was the most prominent lesion, with endothelial cell lining and vascular wall loss and edema uniformly affecting vessels regardless of size (Fig 6B-6F). The spectrum of vasculitis lesions included a progressively increased in density mononuclear cell and macrophage cuffing (Fig 6C-6F). The bronchial tree mucosa showed multifocal sub-epithelial edema, and degeneration, necrosis, and desquamation of epithelial cells (Fig 6C-6D). There was diffuse, prominent hyaline membrane formation and focal micro-hemorrhages in the alveolar septa. Multifocally, alvelolar spaces were filled with variable amounts of protein-rich edematous fluid, fibrin and relatively small numbers of necrotic cell debris and alveolar macrophages (Fig 6D-6E and 6G-6H). Alveolar Type II cell proliferation and formation of syncytial cells were unremarkable. The Martius-Scarlett-Blue (MSB) stain highlighted the extent of hyaline membrane deposition and presence of intra-alveolar fibrin (Fig 6G-6H) and revealed occasional fibrin thrombi in vessels of the lung (Fig 6G) and other tissues examined, including the brain (Fig 6H).

In the trachea, there was severe congestion of the mucosa and lamina propria edema. Lesions in the epithelium included loss of microvilli, intercellular edema and epithelial cell degeneration and necrosis (S2A Fig). Similar findings were found in the nasal mucosa where there was a denser chronic active rhinitis inflammatory cell infiltrate in the lamina propria with intra-epithelial neutrophils and a necrotic-cell rich, suppurative exudate in the nasal cavity lumen (S2B Fig). Noteworthy histopathological lesions in the remaining organs examined included decreased MALT germinal center cellularity (S2C Fig) and multifocal goblet cell hyperplasia (S2D Fig) in the ileum and thymus medulla histiocytosis with large numbers of red blood cell- or hemosiderin-laden macrophages (S2E-S2F Fig).
