# Supplementary material for: Outbreaks of SARS-CoV-2 in naturally infected mink farms: Impact, transmission dynamics, genetic patterns, and environmental contamination
Source: PLoS Pathog. 2021 Sep 7;17(9):e1009883. doi: 10.1371/journal.ppat.1009883 (PMC8448373; doi:10.1371/journal.ppat.1009883)
Supplement: S1 Table — (DOCX) [file ppat.1009883.s002.docx]

| **Protein** | **Ref. position according to acc. NC_045512** | **Sample ID** | **Amino-acid substitution** |
| --- | --- | --- | --- |
| NSP2 | 2659 | A1-A4 | K618N |
| NSP3 | 3230 | B1-B4 | G171C |
|  | 3485 | B1-B4 | A256T |
|  | 4076 | B1-B3 | V453I |
|  | 4992 | A2 | N758T |
|  | 6843 | A1 | S1375F |
|  | 8244 | A1-A4 | Q1842R |
|  | 8408 | A1, A3-A4 | V1897I |
| NSP4 | 9735 | A3 | F394C |
| NSP5 | 10693 | A1-A2 | N214Y |
| NSP8 | 12191 | A1-A4 | V34F |
| ORF3a | 25937 | B1-B4 | H182L |
|  | 26171 | A1, A3-A4 | M260N |
| E | 26442 | A1 | SS67KP |
|  | 26450 |  | R69K |
|  | 26452 |  | V70L |
|  | 26455 |  | P71S |
|  | 26467 |  | V75L |
| M | 26716 | A2 | F65C |
|  | 26728 |  | A69G |
|  | 26730 |  | V70I |
| N | 28500 | A1-A4 | T76I |
|  | 28808 | B1, B3 | G179S |
|  | 28872 | B2, B4 | G200D |
|  | 28881 | A1-A4, B1-B4 | RG203KR |
|  | 29474 | A1-A4 | D401Y |
| NSP12b | 14408 | A1-A4, B1-B4 | P314L |
| NSP13 | 16772 | A2 | N179S |
|  | 16775 |  | Y180C |
| NSP14 | 19480 | B1-B4 | G481C |
| NSP15 | 20465 | A2 | D282G |
