# Supplementary figures and images for: Outbreaks of SARS-CoV-2 in naturally infected mink farms: Impact, transmission dynamics, genetic patterns, and environmental contamination
Source: PLoS Pathog. 2021 Sep 7;17(9):e1009883. doi: 10.1371/journal.ppat.1009883 (PMC8448373; doi:10.1371/journal.ppat.1009883)

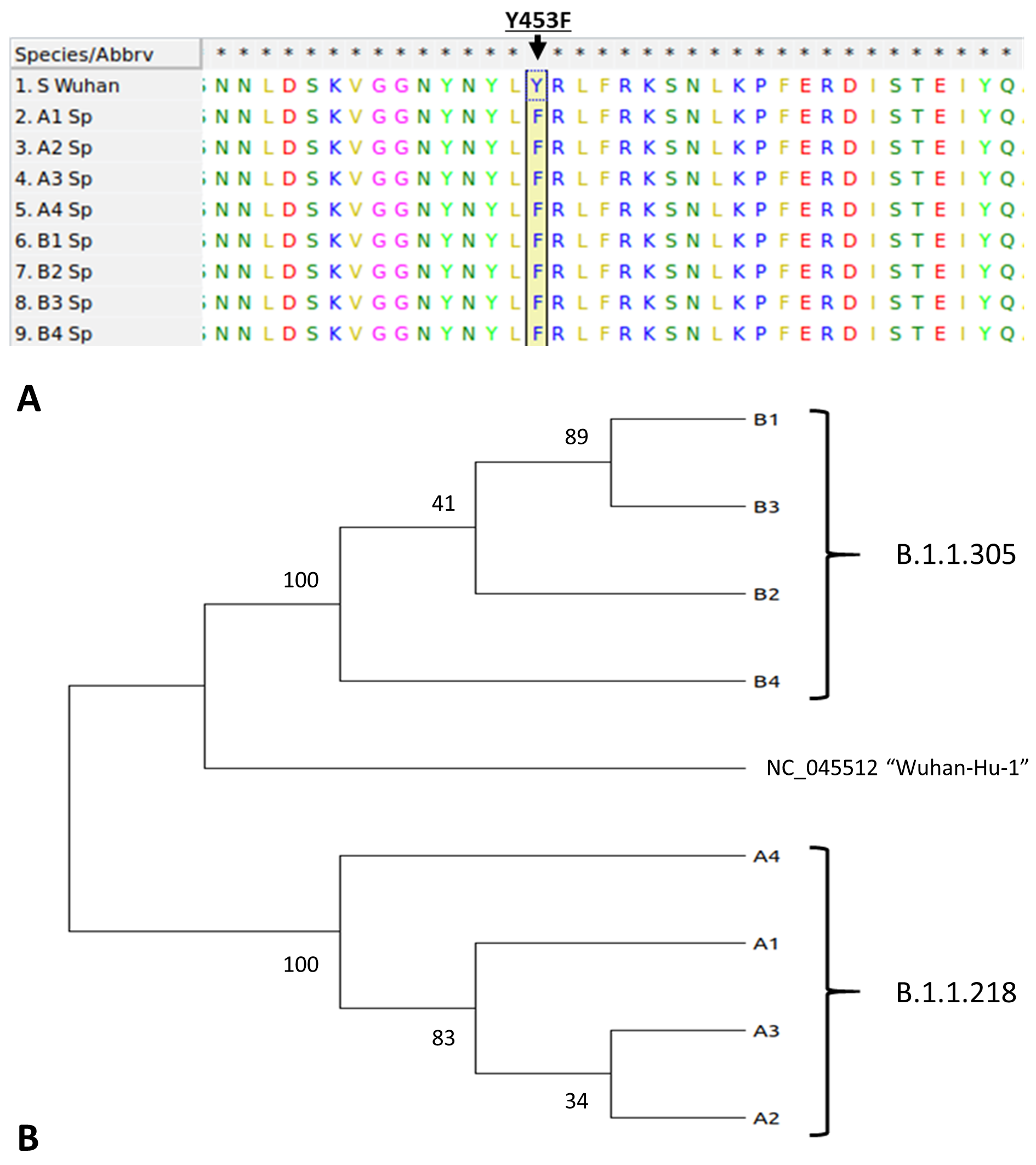

Supplement: S1 Fig — (A) Multiple alignment of the S protein amino-acid sequences with MUSCLE v3.8. The mink-specific Y453F amino-acid substitution is indicated. (B) Phylogenetic analysis of the sequenced samples conducted with MEGA X. The evolutionary history was inferred using the Neighbour-Joining method. The percentage of replicate trees clustered together in the bootstrap test (500 replicates) are shown next to the branches. The evolutionary distances were computed using the Maximum Composite Likelihood method and are in the units of the number of base substitutions per site. The lineage assigned by PANGOLIN is indicated with brackets. (TIF) [file ppat.1009883.s003.tif]

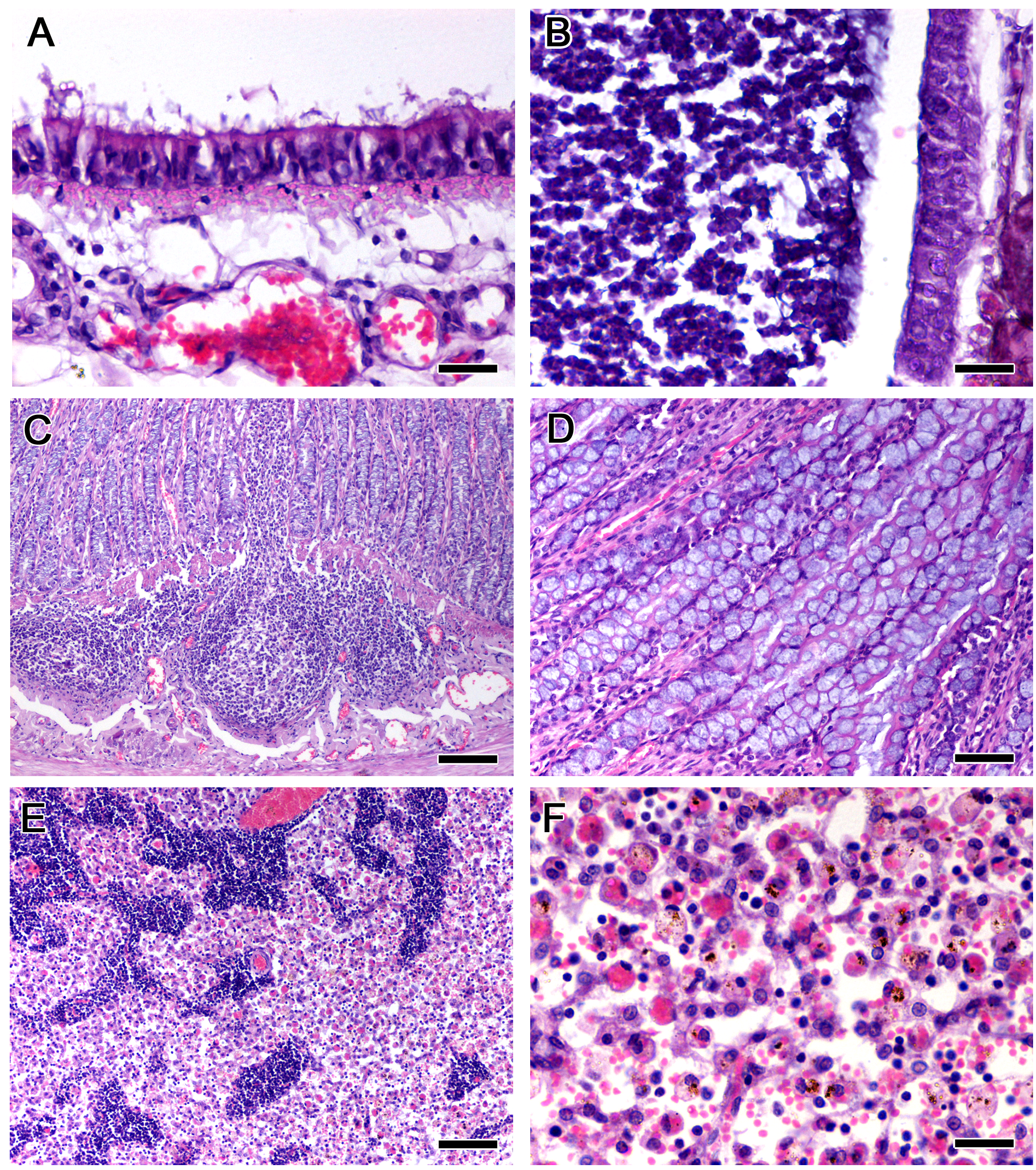

Supplement: S2 Fig — (A) Trachea mucosa with hyperemia, lamina propria edema and respiratory epithelial changes including loss of cilia and degeneration and necrosis of epithelial cells. (B) Nasal conchae respiratory epithelium showing flattening of epithelial cells and loss of cilia, and abundant necrotic-cell rich, suppurative exudate in the cavity. (C and D) Ileum mucosa with loss of germinal center lymphocytes in the submucosal lymphoid tissue (C) and goblet cell hyperplasia (D). (E and F) Thymus medulla histiocytosis (E). There is a large number of macrophages in various stages of red blood cell phagocytosis (F). Hematoxylin and Eosin. Scale bars: 100 μm (C and H); 50 μm (D); 25 μm (A, B and F). (TIF) [file ppat.1009883.s004.tif]
